# Database of Ichthyofauna in urban streams of Johor Bahru, Malaysia

**DOI:** 10.3897/BDJ.13.e148173

**Published:** 2025-03-14

**Authors:** Rei Itsukushima, Mohd Shalahuddin Adnan, Yuta Tomiyama, Yuichi Kano, Keigo Otsu, Muhamad Firdaus Zanorin

**Affiliations:** 1 Kyushu Institute of Technology, Kitakyushu, Japan Kyushu Institute of Technology Kitakyushu Japan; 2 Universiti Tun Hussein Onn Malaysia, Parit Raja, Malaysia Universiti Tun Hussein Onn Malaysia Parit Raja Malaysia; 3 Kumamoto University, Kumamoto, Japan Kumamoto University Kumamoto Japan; 4 Kyushu Open University, Fukuoka, Japan Kyushu Open University Fukuoka Japan; 5 Kyushu University, Fukuoka, Japan Kyushu University Fukuoka Japan

**Keywords:** urban stream, Peninsula Malaysia, fish fauna, mon-native species

## Abstract

**Background:**

Urbanisation leads to the degradation of ecosystems through various factors, such as the deterioration of water quality, changes in water and material cycles and the degradation of biological habitats. Amongst these, aquatic organisms are particularly affected by the loss of habitats due to river canalisation and the impacts of invasive species. It has been widely reported that, in regions where invasive species have been introduced and native species have declined, homogenisation of fish populations occurs, resulting in a significant reduction in biodiversity. This loss of diversity disrupts the ecosystem’s stability and resilience, further compounding the negative effects of urbanisation on aquatic environments. However, the impact of urbanisation on fish populations varies depending on the local ecosystem and the degree of urbanisation, necessitating the examination of ecosystem changes induced by urbanisation in each specific region. The Peninsula Malaysia, which is the focus of this study, is a global hotspot for freshwater biodiversity. However, the effects of urbanisation on fish populations in this region have been scarcely studied. The Masai River Basin, which is the subject of this investigation, is located in the Iskandar Development Region, an area undergoing rapid urbanisation. Understanding the consequences of urbanisation on the fish populations and broader ecosystems in this region is critical for providing information for future conservation and management strategies.

**New information:**

A fish survey was conducted at 19 sites in the Masai River Basin, which is an urbanised watershed, focusing on river channels that have been straightened or converted into concrete-lined waterways. Additionally, fish surveys were conducted at eight sites in non-urbanised areas for comparison. The survey resulted in the collection of nine orders, 15 families, 28 genera, 32 species and a total of 3,007 individuals. In the urbanised sites, the proportion of native species in the total catch was extremely low, averaging only 10.4% across all sites, with invasive species making up the majority of the individuals captured. This indicates the significant shift in species composition due to urbanisation and the dominance of non-native species in these environments. On the other hand, in the non-urbanised areas, the proportion of native species was high at 88.7%, highlighting the significant impact of urbanisation on the invasion of non-native species. Particularly in the downstream areas of the urbanised watershed, species such as *Poeciliasphenops*, *Mayaherosurophthalmus* and *Poeciliareticulata* were frequently captured. In contrast, at sites in the upstream areas where forested habitats remained intact, native species listed on the IUCN Red List, such as *Parambassissiamensis* and *Clariasbatrachus*, were captured. The study revealed that urbanisation and development in the targeted watershed are progressing rapidly, underscoring the urgent need for the conservation and restoration of habitats for these native species.

## Introduction

In monsoon Asia, environmental degradation, such as the intensification of disasters due to climate change and the decline of biodiversity caused by development, is raising concerns about the sustainability of human societies (Sodhi et al. 2009, Loo et al. 2015). Malaysia, the focus of this study, lies entirely within the Sundaland Region and is a global hotspot for freshwater biodiversity, with over 1,000 fish species inhabiting its waters (Giam et al. 2012). However, the rapid and large-scale conversion of tropical rainforests into agricultural land and urban development has led to the degradation of freshwater habitats, resulting in a decline in biodiversity (Chong et al. 2010, Wilkinson et al. 2018). Within Malaysia, while efforts toward biodiversity conservation have advanced in Borneo, the Malay Peninsula, which is the study area, exhibits some of the most significant biodiversity losses in Sundaland and is a priority area for conservation (Chua et al. 2019). This highlights the urgent need for targeted conservation strategies to address the ongoing environmental challenges in this region.

Urban development in Malaysia has been significant, with the urban population in Peninsular Malaysia rising rapidly from 34% in 1980 to 71% in 2010 (Evers 2013). Furthermore, the Malaysian Government set a target to become a high-income nation by 2020 and to achieve an annual GDP growth rate of 6% over the subsequent five years. This economic progress was expected to be accompanied by continued urbanisation (Bekhet and Othman 2017). The rapid pace of urbanisation, coupled with economic growth, is likely to exacerbate environmental challenges, including habitat loss and biodiversity decline, making it essential to implement sustainable development strategies that balance economic growth with ecological conservation. Such development raises concerns about the degradation of river environments.

In recent years, the importance of the ecosystem services provided by urban rivers has been increasingly recognised (Chien and Saito 2021) and the health of urban rivers is now regarded as a crucial element for achieving sustainable cities (Guimarães et al. 2021, Itsukushima and Ohtsuki 2021). For the natural restoration of urban rivers, it is essential to systematically and quantitatively understand the impacts of urbanisation on river systems; however, scientific knowledge in this area remains limited. In particular, in tropical regions, there is a lack of understanding regarding the effects of urbanisation on ecosystems (Yule et al. 2015) and the extent of these impacts remains unclear. Addressing this knowledge gap is vital for the development of effective conservation and restoration strategies for urban river ecosystems.

The Masai River Basin in Johor Bahru, which is the focus of this study, is located within the Iskandar Development Region (Iskandar Malaysia), established in 2006 under the 9^th^ Malaysia Plan. This area is undergoing rapid urbanisation (Yazrin Yasin et al. 2021). As of 2024, more than half of the Basin has been urbanised and development activities continue to progress. This paper reports on the fish survey results from Malaysia’s urban rivers and surrounding areas, where information is scarce. It provides valuable foundational knowledge for environmental conservation and natural restoration of small- and medium-sized urban rivers in tropical regions, contributing to the ongoing efforts for sustainable management in rapidly urbanising areas.

## Sampling methods

### Study extent

**Figure 1.**
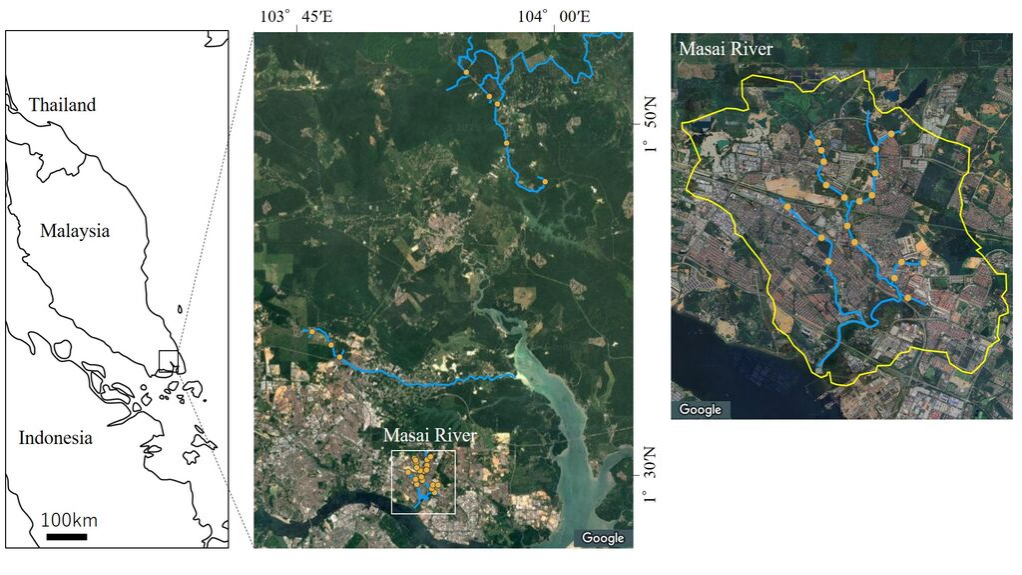
Location of the study sites. The study focused on 27 sites (19 sites belonged to urban area, eight sites belonged to non-urban area).

This survey was conducted in the Masai River Basin, located in the southern part of Johor Bahru, Malaysia, covering 19 sites along urban rivers in the Basin and eight surrounding non-urbanised sites. The Masai River is a 7 km-long main river course that flows into the Johor Straits, with a drainage area of 26 km² (Fig. 1). The Basin is part of the Iskandar Development Region (Iskandar Malaysia), established in 2006 under the 9^th^ Malaysia Plan and has undergone significant urbanisation. Many of the river channels in the area have been modified with concrete linings, raising concerns about the degradation of the river ecosystem. However, there is a lack of information on urbanisation and biodiversity loss in tropical regions. Therefore, a fish survey was conducted to assess the current state of the fish population in the region. Additionally, comparisons were made with rivers not affected by urbanisation. The fish survey was carried out between 28 November 28 and 2 December 2024.

### Sampling description

The fishes were collected by hand nets and throwing nets (cast nets) at each habitat (rapid, run, glide, shallows and pool). For each habitat, approximately 20 net casts (half mesh 5.0 mm, 14.0 m in circumference) and 30 min of sampling with a hand net (500 mm in diameter, 6 mm mesh) were conducted. In this study, a total of 3,007 occurrences were recorded, with species identified both on-site and in the laboratory according to Kottelat 2013, Froese and Pauly (2025) and Naser (2022).

## Geographic coverage

### Description

Surveys were conducted at 19 sites in the Masai River Basin in Johor Bahru, Malaysia, focusing on river channels that have been straightened or converted into concrete channels due to urbanisation. Additionally, surveys were carried out at eight sites in the surrounding non-urbanised watersheds to compare the impact of urbanisation on the fish populations and river ecosystems.

### Coordinates

1.48112 and 1.88235 Latitude; 103.99474 and 103.77058 Longitude.

## Taxonomic coverage

### Description

As a result of the surveys, 15 families, 28 genera, 32 species and 3,007 individuals were collected from the 34 habitats of 27 stations. The highest number of species was 10 (sites belonging to the Tiram River) and the highest number of individuals was 756 (site belonging to the Masai River). By contrast, fish were not confirmed in the site belonging to the Masai River. The highest number of individuals found was 1,447 of *Poeciliasphenops*, which appeared in nine stations.

**Figure 2.**
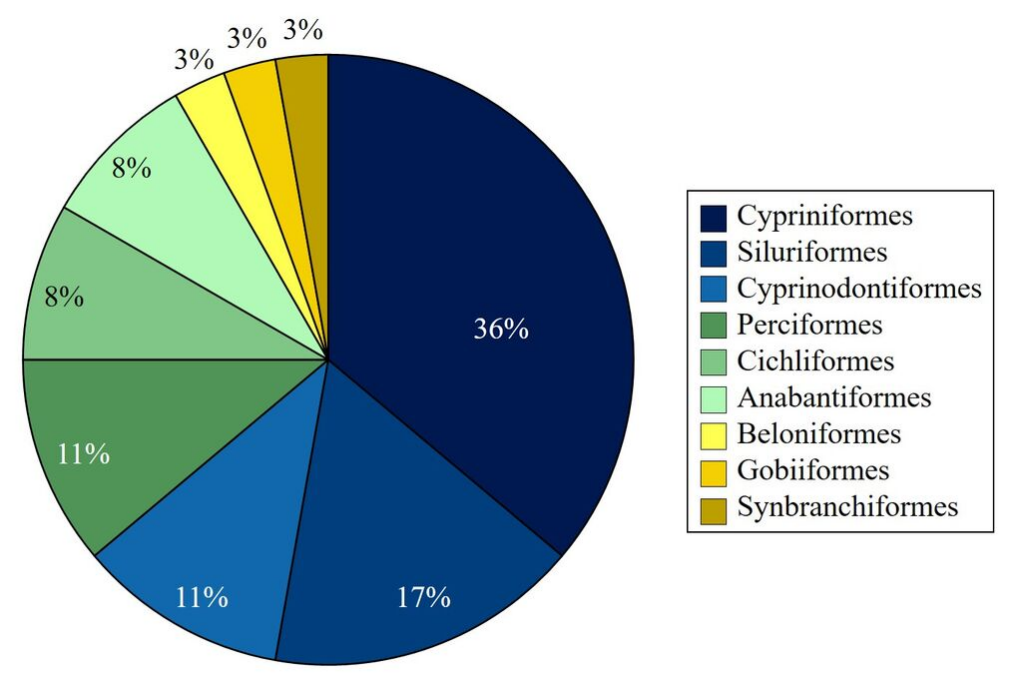
Taxonomic coverage (by order).

**Figure 3.**
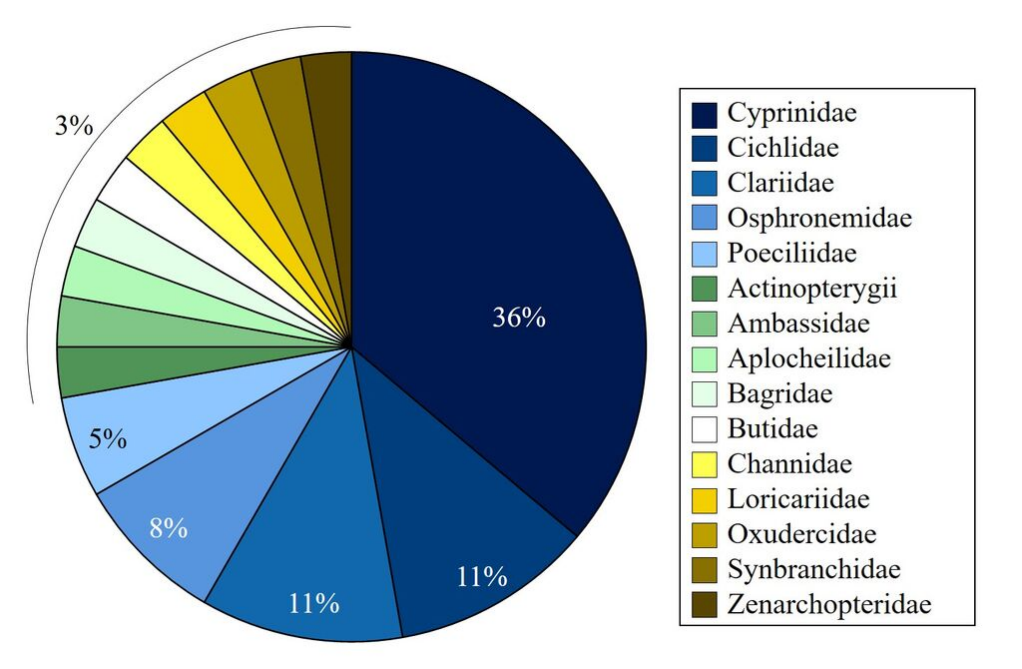
Taxonomic coverage (by family).

The orders were Cypriniformes (13 species), Siluriformes (6 species), Cyprinodontiformes (4 species), Perciformes (4 species), Cichliformes (3 species), Anabantiformes (3 species), Beloniformes (1 species), Gobiiformes (1 species) and Synbranchiformes (1 species) (Fig. 2). The families were Cyprinidae (13 species), Cichlidae (4 species), Clariidae (4 species), Osphronemidae (3 species), Poeciliidae (2 species), Actinopterygii (1 species), Ambassidae (1 species), Aplocheilidae (1 species), Bagridae (1 species), Butidae (1 species), Channidae (1 species), Loricariidae (1 species), Oxudercidae (1 species), Synbranchidae (1 species) and Zenarchopteridae (1 species) (Fig. 3).

Amongst the species confirmed, *Amphilophustrimaculatus* (Günther, 1867), *Geophagussveni* (Lucinda, Lucena & Assis, 2010), *Mayaherosurophthalmus* (Günther, 1862), *Poeciliasphenops* (Valenciennes, 1846), *Gambusiaaffinis* (S. F. Baird & Girard, 1853), *Poeciliareticulata* (W. Peters, 1859), *Puntiuspentazona* (Boulenger, 1894), *Cichlakelberi* (Kullander & Ferreira, 2006), *Clariasgariepinus* (Burchell, 1822) and *Pterygoplichthysdisjunctivus* (Weber, 1991) are considered to be invasive species.

According to the IUCN Red List in 2014, the following species were determined as LC (Least Concern): *Channastriata* (Bloch, 1793), *Bettaimbellis* (Ladiges, 1975), *Trichopsisvittata* (G. Cuvier, 1831), *Dermogenyssiamensis* (Fowler, 1934), *Aplocheiluspanchax* (F. Hamilton, 1822), *Barbodeslateristriga* (Valenciennes, 1842), *Barbodesrhombeus* (Kottelat, 2000), *Cyclocheilichthysapogon* (Valenciennes, 1842), *Danioalbolineatus* (Blyth, 1860), *Esomusmetallicus* (Ahl, 1923), *Parachelamaculicauda* (Smith, 1934), *Rasboraborapetensis* (Smith, 1934), *Rasboradusonensis* (Bleeker, 1850), *Rasboraelegans* (Volz, 1903), *Rasboratrilineata* (Steindachner, 1870), *Parambassissiamensis* (Fowler, 1937), *Oxyeleotrismarmorata* (Bleeker, 1852), *Trichogastertrichopterus* (Pallas, 1770), *Clariasbatrachus* (Linnaeus, 1758), *Clariasnieuhofii* (Valenciennes, 1840) and *Monopterusalbus* (Zuiew, 1793). Additionally, *Labiobarbusfestivus* (Heckel, 1843) is listed as DD (Data Deficient).

### Taxa included

**Table:** 

Rank	Scientific Name	
species	*Channastriata* (Bloch, 1793)	
species	*Bettaimbellis* Ladiges, 1975	
species	*Trichopsisvittata* (G. Cuvier, 1831)	
species	*Dermogenyssiamensis* Fowler, 1934	
species	*Amphilophustrimaculatus* (Günther, 1867)	
species	*Geophagussveni* Lucinda, Lucena & Assis, 2010	
species	*Mayaherosurophthalmus* (Günther, 1862)	
species	*Poeciliasphenops* (Valenciennes, 1846)	
species	*Gambusiaaffinis* (S. F. Baird & Girard, 1853)	
species	*Poeciliareticulata* W. Peters, 1859	
species	*Barbodeslateristriga* (Valenciennes, 1842)	
species	*Barbodesrhombeus* (Kottelat, 2000)	
species	*Cyclocheilichthysapogon* (Valenciennes, 1842)	
species	*Danioalbolineatus* (Blyth, 1860)	
species	*Esomusmetallicus* Ahl, 1923	
species	*Labiobarbusfestivus* (Heckel, 1843)	
genus	Labiobarbus van Hasselt, 1823	
species	*Parachelamaculicauda* (Smith, 1934)	
species	*Puntiuspentazona* (Boulenger, 1894)	
species	*Rasboraborapetensis* Smith, 1934	
species	*Rasboradusonensis* (Bleeker, 1850)	
species	*Rasboraelegans* Volz, 1903	
species	*Rasboratrilineata* Steindachner, 1870	
genus	Brachygobius Bleeker, 1874	
species	*Parambassissiamensis* (Fowler, 1937)	
species	*Oxyeleotrismarmorata* Bleeker, 1852	
species	*Cichlakelberi* Kullander & Ferreira, 2006	
species	*Trichogastertrichopterus* (Pallas, 1770)	
genus	Hemibagrus Valenciennes, 1840	
species	*Clariasbatrachus* (Linnaeus, 1758)	
species	*Clariasgariepinus* Burchell, 1822	
species	*Clariasnieuhofii* Valenciennes, 1840	
genus	Clarias Scopoli, 1777	
species	*Pterygoplichthysdisjunctivus* (Weber, 1991)	
species	*Monopterusalbus* (Zuiew, 1793)	

## Usage licence

### Usage licence

Creative Commons Public Domain Waiver (CC-Zero)

## Data resources

### Data package title

Database of Ichthyofauna in Urban Streams of Johor Bahru, Malaysia

### Resource link

https://ipt.pensoft.net/resource?r=database-ichthyofauna-urban-johor-malaysia

### Number of data sets

1

### Data set 1.

#### Data set name

database-ichthyofauna-urban-johor-malaysia

#### Description

Surveys were conducted at 19 sites in the Masai River Basin in Johor Bahru, Malaysia, focusing on river channels that have been straightened or converted into concrete channels due to urbanisation. Additionally, surveys were carried out at eight sites in the surrounding non-urbanised watersheds to compare the impact of urbanisation on the fish populations and river ecosystems.

**Data set 1.** 

Column label	Column description
occurrenceID	An identifier for the Occurrence.
basisOfRecord	The specific nature of the data record.
samplingProtocol	The names of, references to, or descriptions of the methods or protocols used during an Event.
eventDate	The date-time or interval during which an Event occurred.
scientificName	The full scientific name.
scientificNameAuthorship	The authorship information for the scientificName formatted according to the conventions of the applicable nomenclaturalCode.
kingdom	The full scientific name of the kingdom in which the taxon is classified.
phylum	The full scientific name of the phylum or division in which the taxon is classified.
class	The full scientific name of the class in which the taxon is classified.
order	The full scientific name of the order in which the taxon is classified
family	The full scientific name of the family in which the taxon is classified.
taxonRank	The taxonomic rank of the most specific name in the scientificName as it appears in the original record.
identifiedBy	A list (concatenated and separated) of names of people, groups or organisations who assigned the Taxon to the subject.
recordedBy	A list (concatenated and separated) of the globally unique identifier for the person, people, groups or organisations responsible for recording the original Occurrence.
decimalLatitude	The geographic latitude (in decimal degrees, using the spatial reference system given in geodeticDatum) of the geographic centre of a Location.
decimalLongitude	The geographic longitude (in decimal degrees, using the spatial reference system given in geodeticDatum) of the geographic centre of a Location.
habitat	A category or description of the habitat in which the dwc:Event occurred.
coordinateUncertaintyInMetres	The horizontal distance (in metres) from the given decimalLatitude and decimalLongitude describing the smallest circle containing the whole of the Location.
geodeticDatum	The ellipsoid, geodetic datum or spatial reference system (SRS), upon which the geographic coordinates given in decimalLatitude and decimalLongitude are based.
countryCode	The standard code for the country in which the Location occurs. Recommended best practice is to use ISO 3166-1-alpha-2 country codes.
individualCount	The number of individuals represented present at the time of the Occurrence.
occurrenceStatus	A statement about the presence or absence of a Taxon at a Location.
catalogNumber	A list (concatenated and separated) of previous or alternative fully qualified catalogue numbers or other human-used identifiers for the same Occurrence, whether in the current or any other dataset or collection.
language	A language of the resource. Recommended best practice is to use a controlled vocabulary, such as RFC 4646 [RFC4646].
country	The name of the country or major administrative unit in which the Location occurs. Recommended best practice is to use a controlled vocabulary, such as the Getty Thesaurus of Geographic Names.
stateProvince	The name of the next smallest administrative region than country (state, province, canton, department, region etc.) in which the Location occurs.
municipality	The full, unabbreviated name of the next smallest administrative region than county (city, municipality etc.) in which the Location occurs. Do not use this term for a nearby named place that does not contain the actual location.
waterBody	The name of the water body in which the dcterms:Location occur.
modified	The most recent date-time on which the resource was changed. For Darwin Core, recommended best practice is to use an encoding scheme, such as ISO 8601:2004(E).
year	The four-digit year in which the Event occurred, according to the Common Era Calendar.
month	The ordinal month in which the Event occurred.
day	The integer day of the month on which the Event occurred.
establishmentMeans	Statement about whether a dwc:Organism has been introduced to a given place and time through the direct or indirect activity of modern humans.
dynamicProperties	A list of additional measurements, facts, characteristics or assertions about the record. Meant to provide a mechanism for structured content.

## Additional information

**Figure 4.**
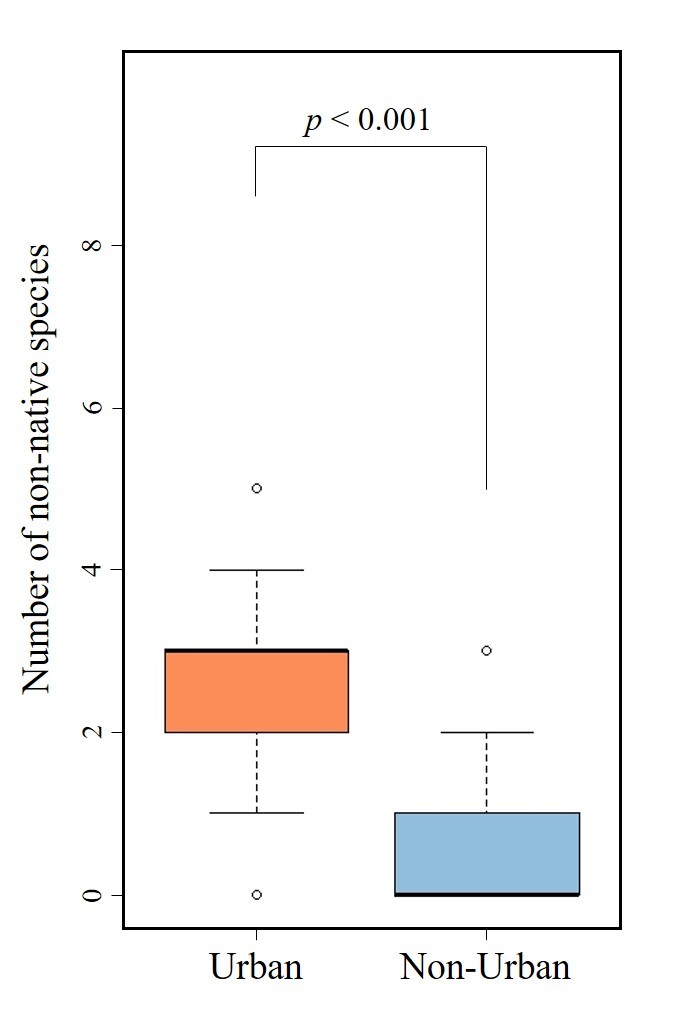
The comparison of the number of non-native species between urban and non-urban areas.

The results of this survey identified 23 species of natives in 18 genera and natives in three genera which were not identifiable to species, for a total of 20 native genera. Further, we identified nine species of introduced fish in eight genera and introduced species in one already-seen genus which was not identifiable to species. The comparison of the number of non-native species between urban and non-urban areas revealed that the urban area had an average of 2.8 ± 1.4 species (mean ± standard deviation), while the non-urban area had 0.6 ± 1.2 species. A significant difference was observed in the results of the t-test (Fig. 4).

Amongst the non-native species, *Poeciliasphenops* was the most abundant, with 1,447 individuals captured in the urban watershed, accounting for approximately 48% of the total number of fish collected in this survey. *Poeciliasphenops* is a species native to Central and South America and from Mexico to Colombia (Ghazi et al. 2019) and is commonly introduced as an ornamental fish (Brito et al. 2013). Known for its high reproductive capacity, a typical characteristic of highly invasive species (Lockwood et al. 2007) and its ability to thrive in turbid environments (Ramírez-García et al. 2017), it is likely that the large number of individuals captured in the urban watershed is a result of these traits.

The next most abundant species captured was *Mayaherosurophthalmus*, with 430 individuals collected. Mayaherosurophthalmus is native to tropical America (Lardizábal et al. 2020) and has expanded its distribution range to Southeast Asia, including Malaysia (Nico 2007, NG et al. 2018). This species is known for its ability to adapt to a wide range of water temperatures and its tolerance to low oxygen conditions (Schofield et al. 2009, Chelapurath Radhakrishnan et al. 2020), suggesting that the urban river environments studied in this research provide a suitable habitat for its survival. Additionally, *Mayaherosurophthalmus* is favoured as a food source in Southeast Asia including the study area (Nico 2007, Ordoñez et al. 2015). On the other hand, *Pterygoplichthysdisjunctivus*, originally native to the Amazon River (Armbruster and Page 2006) and widely distributed in the urban rivers targeted in this study, was first recorded in Malaysia in the early 1990s (Anand Prakash et al. 2022) and is currently expanding its distribution range within the country (Olawale Saba et al. 2024). In the urban rivers surveyed, *Mayaherosurophthalmus* and *Pterygoplichthysdisjunctivus* were confirmed sympatrically, while *Pterygoplichthysdisjunctivus* is typically removed when captured, *Mayaherosurophthalmus* appears to be released back into the river due to its value as a fishery resource. This suggests that the perceived value of these species as food resources may play a significant role in their proliferation in these environments.

Within urban watersheds, the proportion of non-native species also varied. In areas where urbanisation was concentrated downstream, many sites were dominated by non-native species, while in upstream areas with remaining forests, native species listed on the Red List, such as *Parambassissiamensis* and *Clariasbatrachus*, were confirmed. This phenomenon, where rare species emerge from the small remnants of non-urban environments within urban watersheds, has been reported in other climate zones as well (Furlan et al. 2012, Wahlroos et al. 2015, Itsukushima and Maruoka 2022, Itsukushima et al. 2024). The conservation of habitats for these rare species and the restoration of these environments to expand their habitats are critical challenges in the natural regeneration of urban rivers. Future studies that clarify the relationship between physical environments, water quality and fish community structures will help develop environmental restoration methods for urban rivers aimed at the conservation and regeneration of native fish species in tropical urban watersheds.
